# Supplementary material for: Accurate estimation of cell-type composition from gene expression data
Source: Nat Commun. 2019 Jul 5;10:2975. doi: 10.1038/s41467-019-10802-z (PMC6611906; doi:10.1038/s41467-019-10802-z)
Supplement: Supplementary file 1 — Supplementary Information [file 41467_2019_10802_MOESM1_ESM.pdf]

Supplementary Information

Accurate estimation of cell-type composition from gene expression data

Tsoucas et al.

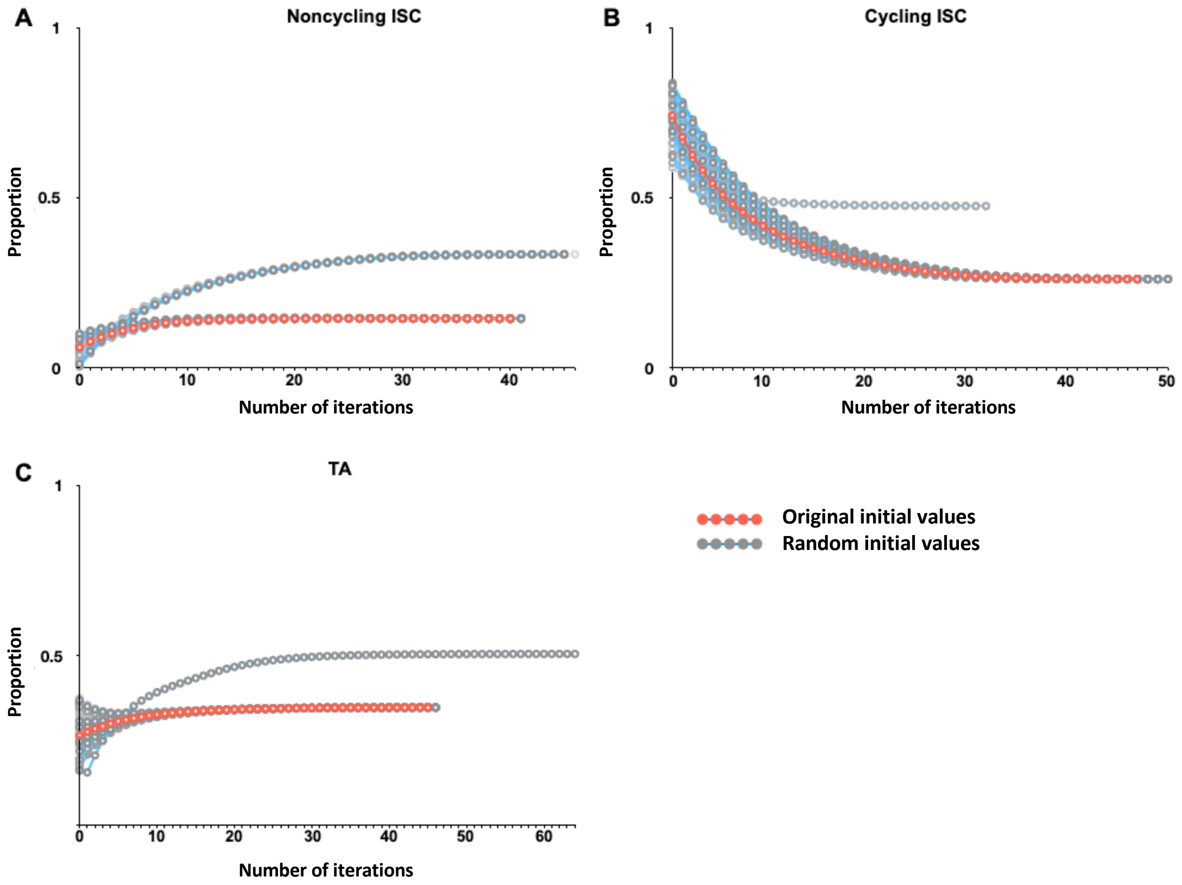

Supplementary Figure 1. Convergence of DWLS. (a) Cell type proportion estimated at each iteration step given 50 random initial values of noncycling ISC, (b) cycling ISC and (c) TA cells from the ISC data set. The red line indicates original initial values. Gray lines indicate randomly selected initial values.

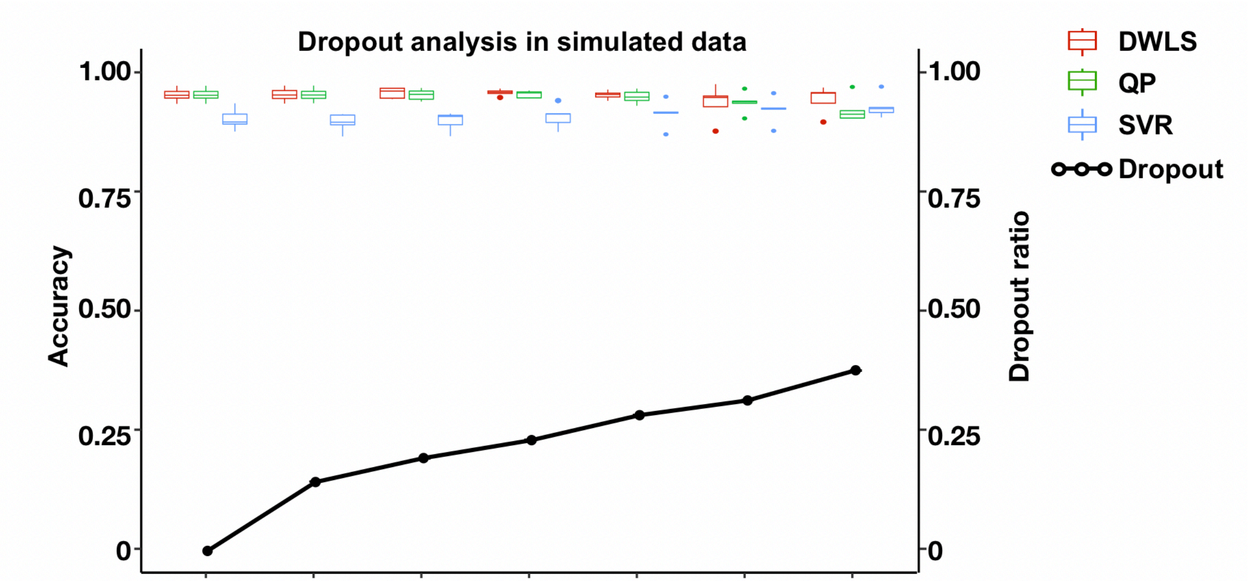

Supplementary Figure 2. Effect of dropout on deconvolution. Accuracy of cell type proportion estimation in simulated data with various levels of dropout, for DWLS, ν-SVR, and QP deconvolution methods. Boxplot indicates cell type proportion prediction accuracy. The centre line of the boxplot corresponds to the median value, while bounds of the boxplot correspond to the 25th and 75th percentiles. The upper whisker bound corresponds to the smaller of the maximum value and the 75th percentile plus 1.5 interquartile ranges; the lower corresponds to the larger of the smallest value and the 25th percentile minus 1.5 interquartile ranges. Point plot indicates percent dropout in each simulated data set.

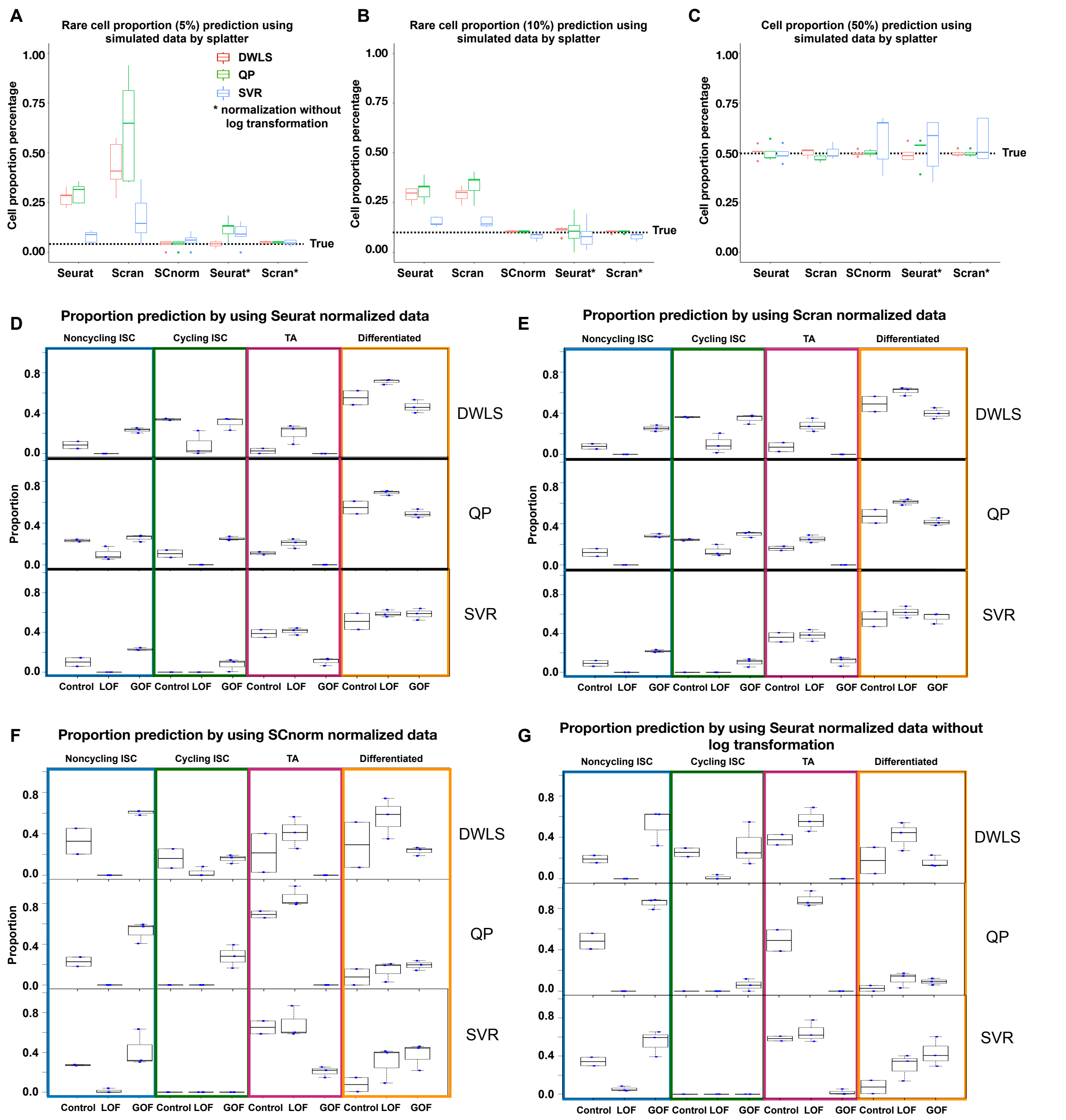

Supplementary Figure 3. Effect of normalization on deconvolution. (a) Rare cell type proportion (5%), (b) rare cell type proportion (10%), and (c) major cell type proportion (50%) estimates using data simulated from Splatter, normalized using Scran (with and without log-transformation), Seurat (with and without log-transformation), and SCnorm, for DWLS, ν-SVR, and QP deconvolution methods. (d) Estimation of cell type proportion on Scran-normalized, (e) Seurat-normalized, (f) SCnorm-normalized, and (g) Seurat-normalized (without log transformation) single-cell data in the ISC data set, for DWLS, ν-SVR, and QP deconvolution methods. The centre line of the boxplot corresponds to the median value, while bounds of the boxplot correspond to the 25th and 75th percentiles. The upper whisker bound corresponds to the smaller of the maximum value and the 75th percentile plus 1.5 interquartile ranges; the lower corresponds to the larger of the smallest value and the 25th percentile minus 1.5 interquartile ranges.

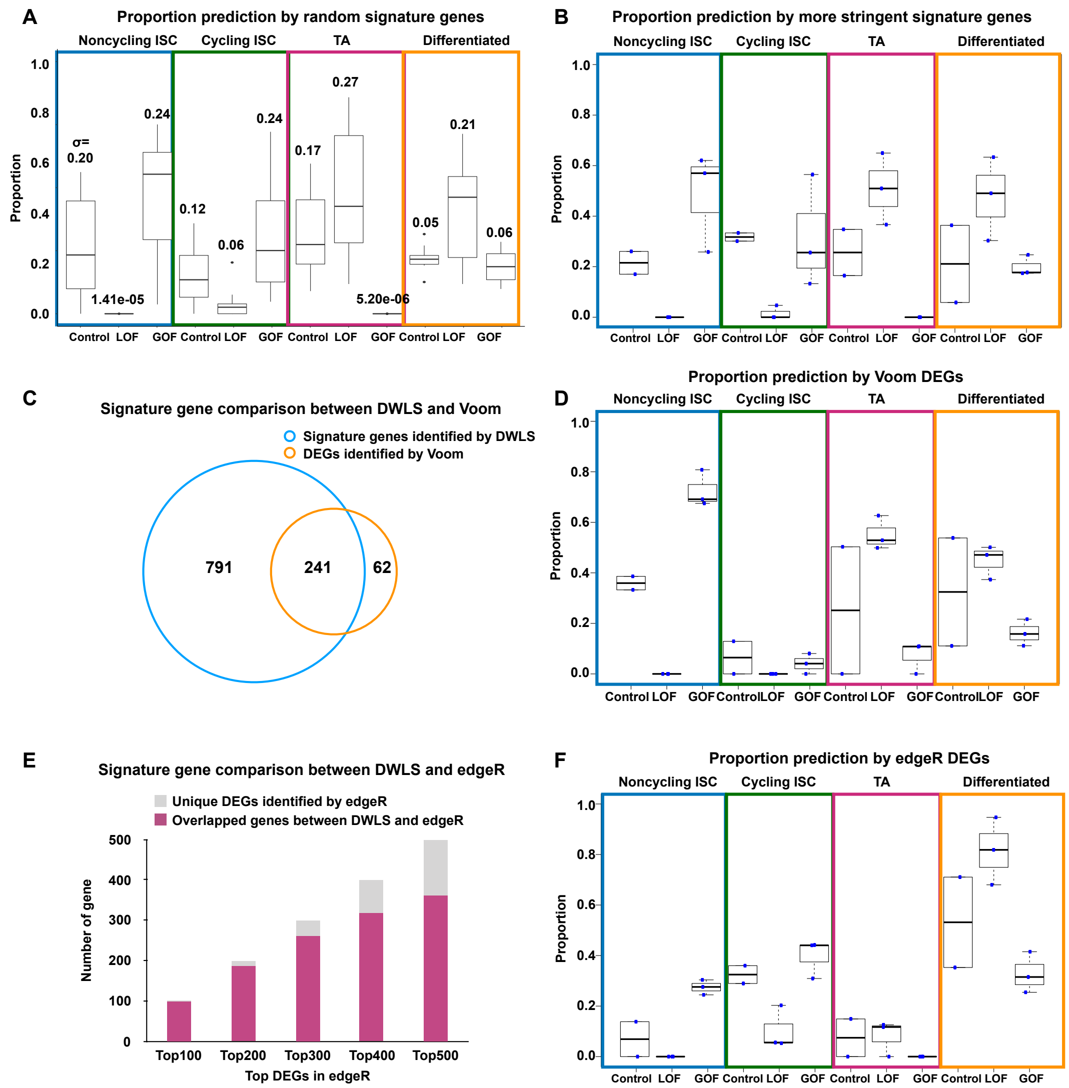

Supplementary Figure 4. Effect of signature gene selection on deconvolution. (a) Cell type proportion estimation by randomly selecting half of signature genes 10 times in ISC data sets. For all boxplots, the centre line of the boxplot corresponds to the median value, while bounds of the boxplot correspond to the 25th and 75th percentiles. The upper whisker bound corresponds to the smaller of the maximum value and the 75th percentile plus 1.5 interquartile ranges; the lower corresponds to the larger of the smallest value and the 25th percentile minus 1.5 interquartile ranges. (b) Cell type proportion estimation using genes with log(Foldchange) ≥ 1 and p-value < 0.001 (FDR adjusted, defined using the hurdle model in the MAST R package) in intestinal stem cells (ISCs). (c) Venn diagram indicates overlapped and specific signature genes identified by DWLS and Voom. (d) Cell type proportion estimation using differentially expressed genes identified by Voom (e) Comparison of signature genes identified by DWLS and edgeR. (f) Cell type proportion estimation using differentially expressed genes identified by edgeR.

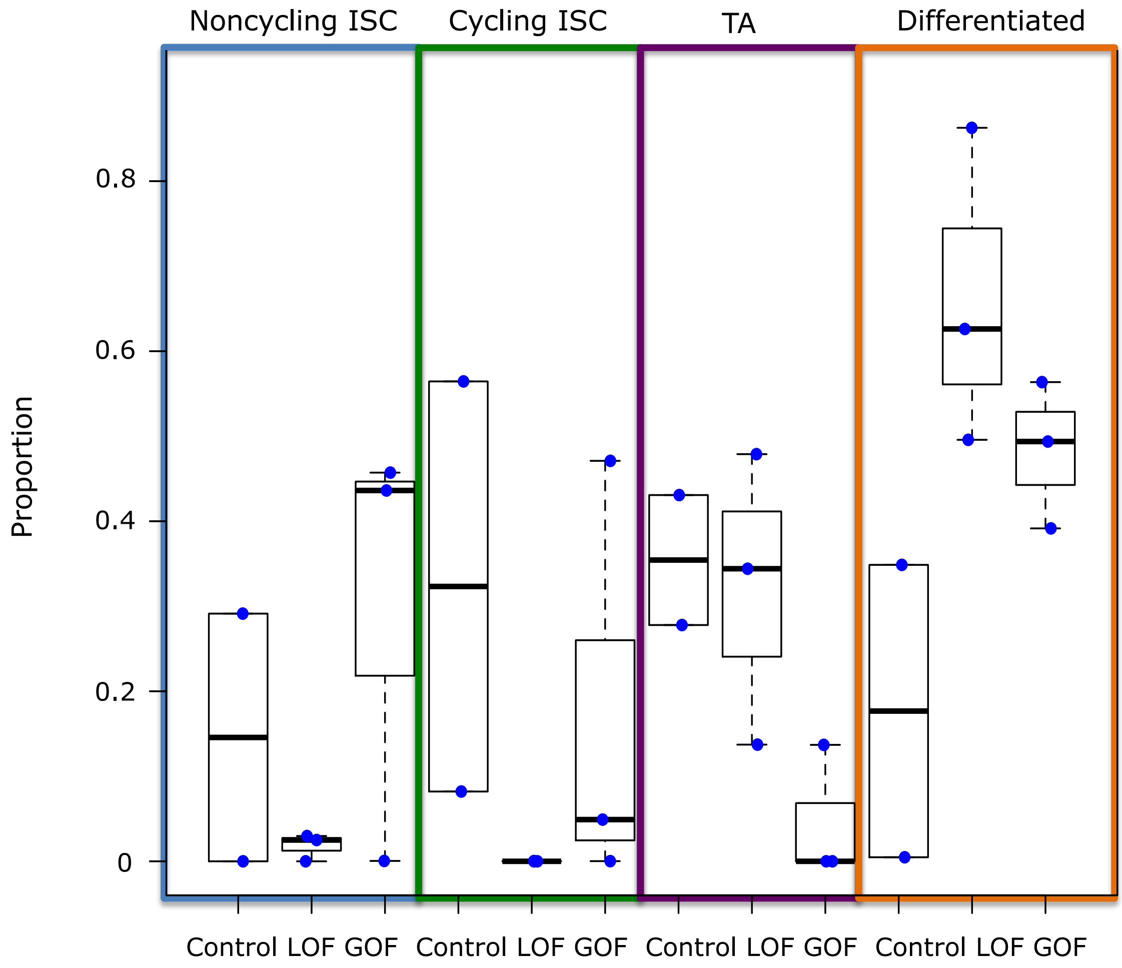

Supplementary Figure 5. Alternate dampening parameter results. Estimated cell type proportion given alternate dampening parameter selection for ISC data, where dampening parameter *d* is selected by minimizing the coefficient of variation.

	T cell (0.439)	B cell (0.098)	Macrophage/ Monocyte (0.150)	Dendritic cell (.010)	NK cell (.032)	Endothelial cell (.008)	Cancer Associated Fibroblast (0.019)	Ovarian cancer cell (.020)	Melanoma cell (0.191)	Overall
DWLS	.086	.039	.044	.004	.027	.008	.024	.023	.029	.032
QP	.075	.030	.045	.008	.040	.019	.057	.019	.058	.038
ν-SVR	.103	.030	.043	.008	.037	.011	.014	.010	.065	.035

Supplementary Table 1. The accuracy of deconvolution results for the 27 simulated bulk data sets. Bulk data is created by summing 27 different donor and patient immune and tumor cell single-cell data sets. Estimation accuracy is measured using absolute error and is calculated for three different deconvolution methods: DWLS, ν-SVR, and QP. Average true proportions for each cell type are listed alongside each cell type name.
